# 3,3′-Bis(quinolin-8-yl)-1,1′-[4,4′-methyl­enebis(4,1-phenyl­ene)]diurea

**DOI:** 10.1107/S1600536811053220

**Published:** 2011-12-17

**Authors:** Avijit Pramanik, Tiffany H. Russ, Douglas R. Powell, Md. Alamgir Hossain

**Affiliations:** a Department of Chemistry and Biochemistry, 1400 J. R. Lynch St, PO Box 17910, Jackson State University, Jackson, MS 39217-0510, USA; b Department of Chemistry and Biochemistry, University of Oklahoma, 620 Parrington Oval, Room 208, Norman, OK 73019-3051, USA

## Abstract

The title compound, C_33_H_26_N_6_O_2_, contains two 3-(quinolin-8-yl)urea groups linked to a diphenyl­methane. The asymmetric unit contains two mol­ecules, *A* and *B*. Each quinoline plane is essentially parallel to the attached urea unit [dihedral angles = 8.97 (18) and 8.81 (19) in molecule *A* and 18.47 (18) and 4.09 (19)° in molecule *B*]. The two benzene rings are twisted, making dihedral angles of 81.36 (8)° in *A* and 87.20 (9)° in *B*. The molecular structures are stabilized by intramolecular N—H⋯N hydrogen bonds. In the crystal, each urea O atom is involved in two N—H⋯O hydrogen bonds, generating two inter­penetrating three-dimensional sets of mol­ecules.

## Related literature

For general background to urea-based compounds in supra­molecular chemistry, see: Fan *et al.* (1993 ▶); Smith *et al.* (1992 ▶); Pramanik *et al.* (2011 ▶); Caltagirone *et al.* (2008 ▶); Custelcean *et al.* (2005 ▶). For related structures, see: Wu *et al.* (2008 ▶); Saeed *et al.* (2010 ▶).
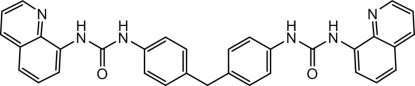


## Experimental

### 

#### Crystal data

C_33_H_26_N_6_O_2_
*M* *_r_* = 538.60
Tetragonal, 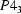

*a* = 18.1345 (6) Å
*c* = 17.1405 (11) Å
*V* = 5636.8 (5) Å^3^
*Z* = 8
Mo *K*α radiation
μ = 0.08 mm^−1^
*T* = 100 K
0.35 × 0.34 × 0.34 mm

#### Data collection

Bruker APEX CCD diffractometer
Absorption correction: multi-scan (*SADABS*; Sheldrick, 2001 ▶) *T*_min_ = 0.972, *T*_max_ = 0.973
65350 measured reflections
5737 independent reflections
4589 reflections with *I* > 2σ(*I*)
*R*_int_ = 0.104

#### Refinement

*R*[*F* ^2^ > 2σ(*F* ^2^)] = 0.046
*wR*(*F* ^2^) = 0.104
*S* = 1.00
5737 reflections
767 parameters
1 restraint
H atoms treated by a mixture of independent and constrained refinement
Δρ_max_ = 0.16 e Å^−3^
Δρ_min_ = −0.17 e Å^−3^

### 

Data collection: *SMART* (Bruker, 2007 ▶); cell refinement: *SAINT* (Bruker, 2007 ▶); data reduction: *SAINT*; program(s) used to solve structure: *SHELXTL* (Sheldrick, 2008 ▶); program(s) used to refine structure: *SHELXTL*; molecular graphics: *SHELXTL*; software used to prepare material for publication: *SHELXTL*.

## Supplementary Material

Crystal structure: contains datablock(s) global, I. DOI: 10.1107/S1600536811053220/rk2320sup1.cif

Structure factors: contains datablock(s) I. DOI: 10.1107/S1600536811053220/rk2320Isup2.hkl

Additional supplementary materials:  crystallographic information; 3D view; checkCIF report

## Figures and Tables

**Table 1:** Hydrogen-bond geometry (Å, °)

*D*—H⋯*A*	*D*—H	H⋯*A*	*D*⋯*A*	*D*—H⋯*A*
N11*A*—H11*A*⋯N1*A*	0.74 (4)	2.27 (4)	2.625 (4)	110 (3)
N11*A*—H11*A*⋯O13*A*^i^	0.74 (4)	2.41 (4)	3.101 (3)	155 (4)
N14*A*—H14*A*⋯O13*A*^i^	0.86 (4)	1.97 (4)	2.810 (4)	166 (3)
N28*A*—H28*A*⋯O30*A*^ii^	0.78 (4)	2.05 (4)	2.827 (4)	170 (4)
N31*A*—H31*A*⋯O30*A*^ii^	0.88 (4)	2.56 (4)	3.293 (4)	141 (3)
N31*A*—H31*A*⋯N39*A*	0.88 (4)	2.14 (4)	2.635 (4)	114 (3)
N11*B*—H11*B*⋯N1*B*	0.90 (4)	2.13 (4)	2.645 (4)	116 (3)
N11*B*—H11*B*⋯O30*B*^iii^	0.90 (4)	2.46 (4)	3.172 (4)	136 (3)
N14*B*—H14*B*⋯O30*B*^iii^	0.80 (3)	1.98 (4)	2.772 (4)	167 (3)
N28*B*—H28*B*⋯O13*B*^iv^	0.87 (4)	1.94 (4)	2.786 (4)	161 (4)
N31*B*—H31*B*⋯O13*B*^iv^	0.90 (4)	2.36 (3)	3.115 (4)	141 (3)
N31*B*—H31*B*⋯N39*B*	0.90 (4)	2.14 (3)	2.647 (4)	115 (3)

Symmetry codes: (i) 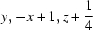; (ii) 
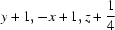
; (iii) 
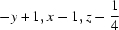
; (iv) 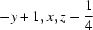.

**Fig. 1.**
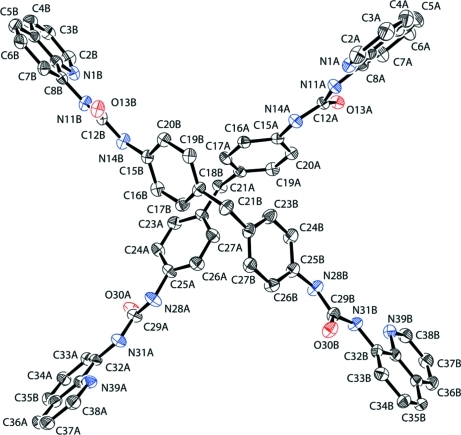
The molecular structure of the title compound showing two molecules (A and B) with the atom-numbering scheme. The hydrogen atoms have been omitted clarity. Displacement ellipsoids are drawn at the 50% probability level.

**Fig. 2.**
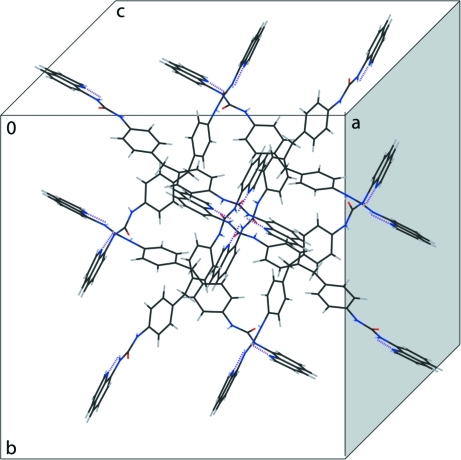
Packing structure of title compound viewed along c axis.

## supplementary crystallographic information

### Comment

In supramolecular chemistry, urea-based compounds are known to effectively bind anions in which a urea group acts as H-bond donors. For examples, acyclic urea hosts containing one or two binding sites were able to form complexes with phosphonates, sulfates and carboxylates in CHCl_3_ (Smith *et al.*, 1992) or acetate and glutarate in *DMSO* (Fan *et al.*, 1993). Tren-based urea receptors with three urea units were recently reported showing high affinity and selectivity for various inorganic anions (Custelcean *et al.*, 2005; Wu *et al.*, 2008; Caltagirone *et al.*, 2008). In an earlier paper, we reported a seven-coordinated hydrogen sulfate formed with three tren-based ureas *via* six NH···O bonds (*d*_N···O_ = 2.85-3.09Å) and one OH···O bond (*d*_O···O_ = 2.57Å) (Pramanik *et al.*, 2011). In an effort to design selective receptors with a rigid framework, we synthesized a dipodal receptor consisting of a diphenylmethane linked with two quinoline groups. The title *bis*-urea compound contains two urea binding sites that could be an effective receptor for binding of variety of anions.

The *bis*-urea receptor crystallized in the tetragonal space group *P*4_3_ with two molecules (Fig. 1) in the asymmetric unit. The asymmetric unit contains two molecules - A and B. As shown in Fig. 1, the two carbonyls of the two urea fragments of each molecule are oriented in the same direction. Two phenyl rings are twisted giving dihedral angles of 81.36 (8)° for A and 87.20 (9)° for B. Each pyridine nitrogen of the quinoline groups is involved in strong intramolecular hydrogen bonding with one NH group with N···N distances ranging from 2.625 (4)Å to 2.647 (4)Å. Each quinoline plane is nearly parallel with the attached urea group. There was no intermolecular hydrogen bonding between the two molecules. Each oxygen atom is bonded with two intermolecular NH···O hydrogen bonds with N···O distances ranging from 2.772 (4)Å to 3.293 (4)Å. Similar H-bonding interactions were observed in a related bis urea receptor (Saeed *et al.*, 2010). In the extended structure viewed along the *c* axis, quinoline planes are found to be antiparallel (Fig. 2). No π···π stacking was observed between the aromatic groups.

### Experimental

Synthesis of 1: 4,4'-methylenebis(phenylisocyanate) (500 mg, 1.99 mmol) was reacted with 8-aminoquinoline (576 mg, 3.99 mmol) in dichloromethane (500 ml) at room temperature under constant stirring. The mixture was refluxed for 5 h. A white precipitate was formed which collected by filtration. The precipitate washed by dichloromethane in several times and dried under vacuum to give a white solid (1.022 g, 95% yield). *δ*_H_ (500 MHz; *DMSO*-*d_6_*) 9.80 (2*H*, s, *Ar*–N*H*), 9.66 (2*H*, s, *Ar*–N*H*), 8.91 (2*H*, d, J = 4.15 Hz, *Ar*H), 8.54 (2*H*, dd, J1 = 3.1 Hz, J2 = 3.25 Hz, J3 = 3.55 Hz, *Ar*H), 8.38 (2*H*, d, J = 8.25 Hz, *Ar*H), 7.62 (2*H*, dd, J1 = 4.15 Hz, J2 = 3.95 Hz, J3 = 4.2 Hz, *Ar*H) 7.55 (2*H*, d, J = 2 Hz, *Ar*H), 7.54 (2*H*, d, J = 0.55 Hz, *Ar*H), 7.41 (4*H*, d, J = 7.6 Hz, *Ar*H), 7.15 (2*H*, d, J = 5.75 Hz, *Ar*H) 3.84 (4*H*, s, αH). *δ*_C_ (125 MHz; *DMSO*-*d_6_*_)_δ 152.4 (CO), 148.3 (C*Ar*), 137.744 (CH*Ar*), 137.7 (CH*Ar*), 136.616 (CH*Ar*), 135.9 (CH*Ar*), 135.1 (CH*Ar*), 129.0 (CH*Ar*), 127.907 (CH*Ar*), 127.2 (CH*Ar*), 122.018 (CH*Ar*), 119.7 (CH*Ar*), 118.3 (CH*Ar*), 114.3 (CH*Ar*). ESI-MS(+ve): m/*z* 539.2 (MH^+^).

### Refinement

H atoms bonded to carbons were positioned geometrically and refined using a riding model with C–H = 0.99Å, *U*_iso_(H) = 1.2*U*_eq_(C). H atoms bonded to N atoms were located on a difference map and their positions were refined independently with *U*_iso_(H) = 1.2*U*_eq_(N).

### Crystal data

**Table:**

C_33_H_26_N_6_O_2_	*D*_x_ = 1.269 Mg m^−^^3^
*M**_r_* = 538.60	Mo *K*α radiation, λ = 0.71073 Å
Tetragonal, *P*4_3_	Cell parameters from 5315 reflections
Hall symbol: P 4cw	θ = 2.3–25.6°
*a* = 18.1345 (6) Å	µ = 0.08 mm^−^^1^
*c* = 17.1405 (11) Å	*T* = 100 K
*V* = 5636.8 (5) Å^3^	Prism, colourless
*Z* = 8	0.35 × 0.34 × 0.34 mm
*F*(000) = 2256	

### Data collection

**Table:**

Bruker APEX CCD diffractometer	5737 independent reflections
Radiation source: fine-focus sealed tube	4589 reflections with *I* > 2σ(*I*)
graphite	*R*_int_ = 0.104
φ and ω scans	θ_max_ = 26.0°, θ_min_ = 1.6°
Absorption correction: multi-scan (*SADABS*; Sheldrick, 2001)	*h* = −21→22
*T*_min_ = 0.972, *T*_max_ = 0.973	*k* = −22→22
65350 measured reflections	*l* = −21→21

### Refinement

**Table:**

Refinement on *F*^2^	Primary atom site location: structure-invariant direct methods
Least-squares matrix: full	Secondary atom site location: difference Fourier map
*R*[*F*^2^ > 2σ(*F*^2^)] = 0.046	Hydrogen site location: inferred from neighbouring sites
*wR*(*F*^2^) = 0.104	H atoms treated by a mixture of independent and constrained refinement
*S* = 1.00	*w* = 1/[σ^2^(*F*_o_^2^) + (0.056*P*)^2^ + 0.2*P*] where *P* = (*F*_o_^2^ + 2*F*_c_^2^)/3
5737 reflections	(Δ/σ)_max_ = 0.009
767 parameters	Δρ_max_ = 0.16 e Å^−^^3^
1 restraint	Δρ_min_ = −0.17 e Å^−^^3^

### Fractional atomic coordinates and isotropic or equivalent isotropic displacement parameters (Å^2^)

**Table:**

	*x*	*y*	*z*	*U*_iso_*/*U*_eq_	
N1A	0.46023 (16)	0.60598 (15)	1.03005 (16)	0.0295 (7)	
C2A	0.4337 (2)	0.6382 (2)	1.0937 (2)	0.0376 (9)	
H2A	0.4386	0.6130	1.1420	0.045*	
C3A	0.3988 (2)	0.7074 (2)	1.0939 (2)	0.0417 (10)	
H3A	0.3812	0.7282	1.1412	0.050*	
C4A	0.3906 (2)	0.7445 (2)	1.0250 (2)	0.0403 (10)	
H4A	0.3664	0.7910	1.0239	0.048*	
C5A	0.4152 (2)	0.7487 (2)	0.8829 (2)	0.0397 (10)	
H5A	0.3911	0.7950	0.8781	0.048*	
C6A	0.4468 (2)	0.7169 (2)	0.8194 (2)	0.0397 (9)	
H6A	0.4449	0.7418	0.7707	0.048*	
C7A	0.4820 (2)	0.6482 (2)	0.8240 (2)	0.0344 (9)	
H7A	0.5034	0.6270	0.7786	0.041*	
C8A	0.48556 (18)	0.61166 (18)	0.89402 (19)	0.0262 (8)	
C9A	0.45352 (19)	0.64369 (19)	0.96179 (19)	0.0281 (8)	
C10A	0.41827 (19)	0.71349 (19)	0.9554 (2)	0.0327 (9)	
N11A	0.51799 (16)	0.54288 (16)	0.90650 (15)	0.0250 (7)	
H11A	0.510 (2)	0.5245 (19)	0.944 (2)	0.030*	
C12A	0.55774 (18)	0.50051 (18)	0.85636 (18)	0.0238 (7)	
O13A	0.57356 (12)	0.51987 (13)	0.78897 (12)	0.0271 (5)	
N14A	0.57950 (16)	0.43545 (16)	0.88795 (16)	0.0265 (7)	
H14A	0.5608 (19)	0.4245 (19)	0.932 (2)	0.032*	
C15A	0.61679 (17)	0.37771 (18)	0.85041 (18)	0.0223 (7)	
C16A	0.60452 (18)	0.30632 (18)	0.87673 (19)	0.0262 (8)	
H16A	0.5719	0.2983	0.9193	0.031*	
C17A	0.63877 (19)	0.24690 (19)	0.84222 (19)	0.0291 (8)	
H17A	0.6287	0.1985	0.8607	0.035*	
C18A	0.68806 (18)	0.25667 (18)	0.78052 (18)	0.0259 (8)	
C19A	0.70224 (19)	0.32855 (19)	0.75691 (19)	0.0295 (8)	
H19A	0.7366	0.3366	0.7159	0.035*	
C20A	0.66824 (18)	0.38898 (19)	0.79072 (18)	0.0264 (8)	
H20A	0.6797	0.4375	0.7736	0.032*	
C21A	0.7215 (2)	0.19087 (19)	0.7397 (2)	0.0322 (8)	
H21A	0.7509	0.2085	0.6948	0.039*	
H21B	0.6813	0.1597	0.7190	0.039*	
C22A	0.77037 (18)	0.14410 (19)	0.79122 (18)	0.0258 (8)	
C23A	0.77950 (19)	0.07013 (19)	0.77598 (19)	0.0312 (8)	
H23A	0.7524	0.0485	0.7344	0.037*	
C24A	0.82653 (19)	0.02622 (19)	0.81896 (19)	0.0312 (8)	
H24A	0.8308	−0.0249	0.8076	0.037*	
C25A	0.86764 (19)	0.05729 (19)	0.87904 (19)	0.0299 (8)	
C26A	0.8583 (2)	0.1311 (2)	0.8964 (2)	0.0347 (9)	
H26A	0.8852	0.1526	0.9381	0.042*	
C27A	0.8099 (2)	0.1739 (2)	0.8534 (2)	0.0335 (9)	
H27A	0.8035	0.2244	0.8665	0.040*	
N28A	0.91573 (19)	0.01542 (18)	0.92631 (17)	0.0349 (8)	
H28A	0.916 (2)	0.028 (2)	0.970 (2)	0.042*	
C29A	0.95213 (19)	−0.0459 (2)	0.9045 (2)	0.0312 (8)	
O30A	0.95297 (14)	−0.06992 (14)	0.83709 (13)	0.0365 (6)	
N31A	0.98804 (16)	−0.08013 (17)	0.96440 (17)	0.0306 (7)	
H31A	0.988 (2)	−0.060 (2)	1.011 (2)	0.037*	
C32A	1.02455 (18)	−0.14765 (19)	0.9634 (2)	0.0263 (8)	
C33A	1.03786 (19)	−0.1903 (2)	0.8987 (2)	0.0319 (8)	
H33A	1.0230	−0.1733	0.8486	0.038*	
C34A	1.0736 (2)	−0.2591 (2)	0.9060 (2)	0.0354 (9)	
H34A	1.0824	−0.2880	0.8607	0.042*	
C35A	1.0956 (2)	−0.2849 (2)	0.9770 (2)	0.0366 (9)	
H35A	1.1195	−0.3313	0.9808	0.044*	
C36A	1.1021 (2)	−0.2668 (2)	1.1212 (2)	0.0392 (10)	
H36A	1.1251	−0.3133	1.1288	0.047*	
C37A	1.0870 (2)	−0.2225 (2)	1.1831 (2)	0.0400 (10)	
H37A	1.0994	−0.2379	1.2344	0.048*	
C38A	1.0530 (2)	−0.1537 (2)	1.1706 (2)	0.0381 (9)	
H38A	1.0432	−0.1236	1.2147	0.046*	
N39A	1.03389 (16)	−0.12856 (17)	1.10094 (17)	0.0329 (7)	
C40A	1.04785 (18)	−0.17355 (18)	1.0386 (2)	0.0274 (8)	
C41A	1.08295 (19)	−0.2426 (2)	1.0450 (2)	0.0332 (9)	
N1B	0.37326 (16)	−0.06095 (15)	0.81946 (16)	0.0295 (7)	
C2B	0.3446 (2)	−0.08341 (19)	0.7527 (2)	0.0340 (9)	
H2B	0.3718	−0.0753	0.7062	0.041*	
C3B	0.2751 (2)	−0.1190 (2)	0.7470 (2)	0.0370 (9)	
H3B	0.2568	−0.1340	0.6975	0.044*	
C4B	0.2352 (2)	−0.1314 (2)	0.8121 (2)	0.0348 (9)	
H4B	0.1888	−0.1554	0.8088	0.042*	
C5B	0.2250 (2)	−0.1194 (2)	0.9571 (2)	0.0331 (8)	
H5B	0.1788	−0.1440	0.9578	0.040*	
C6B	0.2555 (2)	−0.09456 (19)	1.0241 (2)	0.0344 (9)	
H6B	0.2296	−0.1019	1.0717	0.041*	
C7B	0.32440 (19)	−0.05801 (18)	1.0262 (2)	0.0285 (8)	
H7B	0.3444	−0.0416	1.0744	0.034*	
C8B	0.36206 (19)	−0.04658 (18)	0.95747 (19)	0.0256 (8)	
C9B	0.33188 (19)	−0.07224 (18)	0.88527 (19)	0.0266 (8)	
C10B	0.26309 (19)	−0.10829 (18)	0.8857 (2)	0.0290 (8)	
N11B	0.43079 (16)	−0.01080 (16)	0.95094 (17)	0.0285 (7)	
H11B	0.448 (2)	−0.014 (2)	0.902 (2)	0.039 (11)*	
C12B	0.4623 (2)	0.03466 (18)	1.00490 (19)	0.0273 (8)	
O13B	0.43275 (13)	0.05017 (13)	1.06738 (13)	0.0313 (6)	
N14B	0.52838 (17)	0.06201 (16)	0.98239 (18)	0.0292 (7)	
H14B	0.5448 (18)	0.0504 (18)	0.941 (2)	0.019 (9)*	
C15B	0.56730 (19)	0.11839 (18)	1.02099 (19)	0.0253 (7)	
C16B	0.6440 (2)	0.11995 (19)	1.01518 (19)	0.0303 (8)	
H16B	0.6692	0.0824	0.9873	0.036*	
C17B	0.6831 (2)	0.17647 (19)	1.0502 (2)	0.0310 (8)	
H17B	0.7354	0.1769	1.0462	0.037*	
C18B	0.64819 (18)	0.23261 (19)	1.09119 (19)	0.0271 (8)	
C19B	0.57124 (19)	0.23141 (19)	1.09414 (19)	0.0281 (8)	
H19B	0.5459	0.2697	1.1209	0.034*	
C20B	0.53112 (19)	0.17585 (18)	1.05906 (18)	0.0283 (8)	
H20B	0.4788	0.1768	1.0609	0.034*	
C21B	0.69101 (19)	0.2921 (2)	1.1334 (2)	0.0327 (9)	
H21C	0.7051	0.2733	1.1855	0.039*	
H21D	0.6583	0.3351	1.1413	0.039*	
C22B	0.76001 (19)	0.31761 (19)	1.0915 (2)	0.0302 (8)	
C23B	0.7541 (2)	0.3536 (2)	1.0195 (2)	0.0376 (9)	
H23B	0.7072	0.3590	0.9956	0.045*	
C24B	0.8160 (2)	0.3813 (2)	0.9832 (2)	0.0360 (9)	
H24B	0.8113	0.4053	0.9342	0.043*	
C25B	0.88455 (19)	0.37463 (19)	1.01684 (19)	0.0304 (8)	
C26B	0.8918 (2)	0.3362 (2)	1.08675 (19)	0.0321 (8)	
H26B	0.9390	0.3292	1.1097	0.038*	
C27B	0.8290 (2)	0.30840 (19)	1.1222 (2)	0.0318 (8)	
H27B	0.8341	0.2819	1.1697	0.038*	
N28B	0.94570 (17)	0.40586 (18)	0.97736 (18)	0.0346 (8)	
H28B	0.937 (2)	0.417 (2)	0.929 (2)	0.049 (12)*	
C29B	1.0048 (2)	0.4385 (2)	1.0114 (2)	0.0307 (8)	
O30B	1.01626 (14)	0.43771 (14)	1.08229 (13)	0.0360 (6)	
N31B	1.05041 (16)	0.47147 (16)	0.95887 (17)	0.0284 (7)	
H31B	1.0378 (17)	0.4754 (17)	0.908 (2)	0.025 (9)*	
C32B	1.11753 (19)	0.50756 (18)	0.9727 (2)	0.0278 (8)	
C33B	1.1492 (2)	0.5185 (2)	1.0449 (2)	0.0340 (9)	
H33B	1.1248	0.5019	1.0906	0.041*	
C34B	1.2180 (2)	0.55449 (19)	1.0508 (2)	0.0353 (9)	
H34B	1.2392	0.5619	1.1009	0.042*	
C35B	1.2549 (2)	0.5788 (2)	0.9863 (2)	0.0376 (9)	
H35B	1.3013	0.6025	0.9917	0.045*	
C36B	1.2583 (2)	0.5916 (2)	0.8421 (2)	0.0390 (9)	
H36B	1.3048	0.6156	0.8438	0.047*	
C37B	1.2245 (2)	0.5790 (2)	0.7724 (2)	0.0401 (10)	
H37B	1.2477	0.5932	0.7251	0.048*	
C38B	1.1550 (2)	0.5447 (2)	0.7709 (2)	0.0371 (9)	
H38B	1.1317	0.5376	0.7218	0.045*	
N39B	1.12023 (16)	0.52202 (16)	0.83423 (16)	0.0307 (7)	
C40B	1.15499 (19)	0.53350 (19)	0.9046 (2)	0.0290 (8)	
C41B	1.22385 (19)	0.56873 (19)	0.9114 (2)	0.0316 (8)	

### Atomic displacement parameters (Å^2^)

**Table:**

	*U*^11^	*U*^22^	*U*^33^	*U*^12^	*U*^13^	*U*^23^
N1A	0.0325 (16)	0.0316 (16)	0.0246 (15)	−0.0034 (13)	0.0030 (13)	−0.0037 (14)
C2A	0.041 (2)	0.040 (2)	0.032 (2)	−0.0048 (18)	0.0110 (18)	−0.0078 (18)
C3A	0.043 (2)	0.038 (2)	0.045 (2)	0.0004 (19)	0.018 (2)	−0.015 (2)
C4A	0.041 (2)	0.031 (2)	0.048 (2)	0.0005 (18)	0.017 (2)	−0.0058 (19)
C5A	0.043 (2)	0.028 (2)	0.049 (2)	0.0086 (17)	0.001 (2)	0.0047 (19)
C6A	0.052 (2)	0.034 (2)	0.034 (2)	0.0082 (19)	−0.0014 (19)	0.0050 (18)
C7A	0.046 (2)	0.034 (2)	0.0234 (19)	0.0063 (18)	0.0017 (17)	0.0002 (16)
C8A	0.0278 (19)	0.0247 (19)	0.0262 (18)	0.0015 (15)	−0.0031 (15)	0.0007 (15)
C9A	0.0279 (19)	0.0293 (19)	0.0271 (19)	−0.0001 (15)	0.0036 (15)	0.0002 (16)
C10A	0.028 (2)	0.027 (2)	0.043 (2)	−0.0002 (16)	0.0069 (17)	−0.0069 (17)
N11A	0.0351 (17)	0.0285 (17)	0.0115 (13)	0.0057 (13)	0.0046 (13)	0.0051 (12)
C12A	0.0290 (19)	0.0261 (18)	0.0163 (17)	0.0017 (15)	−0.0036 (15)	0.0017 (15)
O13A	0.0327 (13)	0.0356 (14)	0.0131 (12)	0.0034 (11)	0.0010 (10)	0.0031 (10)
N14A	0.0326 (17)	0.0314 (17)	0.0155 (14)	0.0059 (13)	0.0066 (13)	0.0029 (13)
C15A	0.0224 (17)	0.0299 (19)	0.0146 (16)	0.0017 (14)	0.0003 (14)	−0.0007 (14)
C16A	0.0267 (19)	0.032 (2)	0.0195 (16)	0.0009 (16)	−0.0003 (14)	0.0062 (15)
C17A	0.0329 (19)	0.0282 (19)	0.0261 (18)	0.0031 (15)	−0.0022 (16)	0.0049 (16)
C18A	0.0286 (19)	0.0288 (19)	0.0203 (17)	0.0049 (15)	−0.0069 (15)	−0.0006 (15)
C19A	0.032 (2)	0.037 (2)	0.0202 (17)	0.0007 (16)	0.0033 (15)	−0.0025 (16)
C20A	0.0299 (18)	0.0287 (19)	0.0205 (17)	0.0011 (15)	0.0024 (15)	−0.0008 (15)
C21A	0.039 (2)	0.035 (2)	0.0230 (18)	0.0078 (17)	−0.0076 (16)	−0.0064 (16)
C22A	0.0297 (19)	0.0309 (19)	0.0169 (16)	0.0065 (15)	−0.0008 (15)	0.0012 (15)
C23A	0.035 (2)	0.035 (2)	0.0240 (18)	0.0012 (17)	−0.0029 (16)	−0.0060 (16)
C24A	0.042 (2)	0.028 (2)	0.0237 (18)	0.0075 (17)	−0.0012 (17)	−0.0025 (15)
C25A	0.037 (2)	0.033 (2)	0.0201 (17)	0.0117 (17)	0.0028 (16)	0.0021 (15)
C26A	0.041 (2)	0.036 (2)	0.0270 (19)	0.0038 (18)	−0.0078 (17)	−0.0045 (17)
C27A	0.042 (2)	0.029 (2)	0.030 (2)	0.0064 (17)	−0.0067 (17)	−0.0046 (16)
N28A	0.050 (2)	0.040 (2)	0.0151 (15)	0.0179 (16)	−0.0053 (14)	−0.0041 (14)
C29A	0.034 (2)	0.039 (2)	0.0200 (18)	0.0092 (17)	0.0023 (16)	−0.0011 (16)
O30A	0.0459 (15)	0.0472 (16)	0.0165 (12)	0.0181 (13)	−0.0013 (11)	−0.0019 (12)
N31A	0.0374 (18)	0.0373 (18)	0.0171 (14)	0.0103 (14)	−0.0002 (13)	−0.0008 (14)
C32A	0.0232 (18)	0.0278 (19)	0.0280 (19)	0.0033 (15)	0.0000 (15)	0.0008 (16)
C33A	0.032 (2)	0.037 (2)	0.0272 (19)	0.0034 (16)	0.0045 (16)	0.0049 (17)
C34A	0.037 (2)	0.033 (2)	0.036 (2)	0.0060 (17)	0.0016 (18)	−0.0041 (17)
C35A	0.033 (2)	0.0250 (19)	0.051 (2)	0.0009 (16)	−0.0020 (19)	0.0051 (18)
C36A	0.034 (2)	0.036 (2)	0.047 (2)	−0.0016 (17)	−0.0064 (19)	0.0160 (19)
C37A	0.038 (2)	0.051 (3)	0.031 (2)	−0.0075 (19)	−0.0114 (17)	0.0152 (19)
C38A	0.039 (2)	0.047 (2)	0.029 (2)	−0.0064 (19)	−0.0054 (17)	0.0054 (18)
N39A	0.0308 (17)	0.0439 (19)	0.0240 (15)	−0.0008 (14)	−0.0057 (13)	0.0035 (14)
C40A	0.0236 (18)	0.0294 (19)	0.0292 (19)	−0.0009 (15)	0.0019 (15)	0.0048 (16)
C41A	0.0257 (19)	0.039 (2)	0.034 (2)	−0.0048 (17)	−0.0017 (16)	0.0053 (18)
N1B	0.0409 (18)	0.0243 (16)	0.0234 (15)	0.0026 (13)	−0.0019 (14)	−0.0022 (12)
C2B	0.044 (2)	0.033 (2)	0.0245 (19)	0.0024 (18)	−0.0009 (17)	−0.0058 (16)
C3B	0.046 (2)	0.033 (2)	0.032 (2)	0.0064 (18)	−0.0122 (18)	−0.0096 (17)
C4B	0.032 (2)	0.033 (2)	0.039 (2)	0.0014 (17)	−0.0070 (18)	−0.0085 (17)
C5B	0.031 (2)	0.031 (2)	0.037 (2)	−0.0030 (16)	0.0010 (17)	−0.0002 (17)
C6B	0.036 (2)	0.033 (2)	0.035 (2)	−0.0004 (17)	0.0032 (17)	0.0076 (17)
C7B	0.035 (2)	0.0271 (18)	0.0232 (18)	−0.0023 (16)	0.0004 (16)	0.0013 (15)
C8B	0.030 (2)	0.0234 (18)	0.0233 (18)	0.0036 (15)	−0.0004 (15)	0.0024 (14)
C9B	0.0298 (19)	0.0250 (18)	0.0251 (18)	0.0041 (15)	−0.0007 (15)	−0.0011 (15)
C10B	0.031 (2)	0.0221 (18)	0.034 (2)	0.0035 (15)	−0.0048 (16)	−0.0022 (15)
N11B	0.0359 (18)	0.0329 (17)	0.0167 (15)	−0.0084 (14)	0.0028 (13)	0.0003 (13)
C12B	0.038 (2)	0.0216 (18)	0.0226 (19)	−0.0039 (16)	−0.0006 (16)	0.0016 (15)
O13B	0.0428 (15)	0.0319 (13)	0.0191 (12)	−0.0068 (11)	0.0066 (11)	−0.0041 (10)
N14B	0.0385 (19)	0.0328 (17)	0.0162 (15)	−0.0068 (14)	0.0066 (14)	−0.0058 (13)
C15B	0.0316 (19)	0.0269 (18)	0.0174 (16)	−0.0026 (15)	0.0013 (15)	0.0008 (14)
C16B	0.037 (2)	0.031 (2)	0.0229 (18)	0.0009 (17)	0.0052 (16)	−0.0016 (16)
C17B	0.0274 (19)	0.037 (2)	0.0285 (19)	−0.0028 (16)	0.0025 (16)	0.0019 (17)
C18B	0.0298 (19)	0.034 (2)	0.0175 (16)	−0.0073 (16)	0.0027 (15)	0.0021 (15)
C19B	0.035 (2)	0.0302 (19)	0.0188 (16)	−0.0015 (16)	0.0037 (15)	−0.0016 (15)
C20B	0.032 (2)	0.0323 (19)	0.0209 (17)	−0.0013 (16)	0.0023 (15)	0.0008 (15)
C21B	0.033 (2)	0.036 (2)	0.029 (2)	−0.0087 (17)	0.0023 (16)	−0.0052 (16)
C22B	0.035 (2)	0.031 (2)	0.0252 (18)	−0.0074 (16)	0.0019 (16)	0.0014 (16)
C23B	0.037 (2)	0.044 (2)	0.032 (2)	−0.0118 (18)	−0.0086 (17)	0.0049 (18)
C24B	0.042 (2)	0.045 (2)	0.0207 (18)	−0.0118 (19)	−0.0072 (17)	0.0090 (17)
C25B	0.035 (2)	0.036 (2)	0.0205 (18)	−0.0094 (17)	−0.0004 (16)	0.0003 (16)
C26B	0.030 (2)	0.042 (2)	0.0243 (18)	−0.0020 (17)	−0.0005 (16)	0.0026 (17)
C27B	0.038 (2)	0.035 (2)	0.0224 (18)	−0.0053 (17)	−0.0021 (16)	0.0062 (16)
N28B	0.0391 (19)	0.051 (2)	0.0137 (15)	−0.0162 (16)	−0.0035 (14)	0.0038 (14)
C29B	0.036 (2)	0.033 (2)	0.0234 (19)	−0.0075 (17)	0.0023 (16)	0.0029 (16)
O30B	0.0433 (16)	0.0460 (16)	0.0188 (13)	−0.0135 (12)	−0.0044 (11)	−0.0021 (11)
N31B	0.0299 (17)	0.0401 (18)	0.0151 (15)	−0.0067 (14)	−0.0007 (13)	−0.0021 (13)
C32B	0.0290 (19)	0.0247 (18)	0.0297 (19)	−0.0019 (15)	0.0008 (16)	0.0005 (15)
C33B	0.033 (2)	0.035 (2)	0.033 (2)	0.0024 (17)	−0.0025 (17)	0.0012 (17)
C34B	0.035 (2)	0.034 (2)	0.038 (2)	−0.0064 (17)	−0.0080 (18)	−0.0004 (18)
C35B	0.028 (2)	0.029 (2)	0.056 (3)	−0.0021 (16)	−0.0111 (19)	−0.0011 (19)
C36B	0.028 (2)	0.033 (2)	0.055 (3)	−0.0051 (17)	0.0055 (19)	0.007 (2)
C37B	0.037 (2)	0.043 (2)	0.040 (2)	−0.0006 (18)	0.0123 (19)	0.0117 (19)
C38B	0.036 (2)	0.040 (2)	0.036 (2)	0.0006 (18)	0.0074 (18)	0.0077 (18)
N39B	0.0344 (17)	0.0320 (17)	0.0257 (16)	−0.0013 (13)	0.0058 (14)	0.0023 (13)
C40B	0.029 (2)	0.0231 (19)	0.035 (2)	0.0047 (15)	0.0012 (17)	−0.0010 (16)
C41B	0.0273 (19)	0.026 (2)	0.041 (2)	0.0015 (16)	0.0006 (17)	0.0029 (17)

### Geometric parameters (Å, °)

**Table:**

N1A—C2A	1.327 (4)	N1B—C2B	1.321 (4)
N1A—C9A	1.361 (4)	N1B—C9B	1.370 (4)
C2A—C3A	1.406 (5)	C2B—C3B	1.418 (5)
C2A—H2A	0.9500	C2B—H2B	0.9500
C3A—C4A	1.367 (6)	C3B—C4B	1.350 (5)
C3A—H3A	0.9500	C3B—H3B	0.9500
C4A—C10A	1.411 (5)	C4B—C10B	1.421 (5)
C4A—H4A	0.9500	C4B—H4B	0.9500
C5A—C6A	1.359 (5)	C5B—C6B	1.352 (5)
C5A—C10A	1.398 (5)	C5B—C10B	1.420 (5)
C5A—H5A	0.9500	C5B—H5B	0.9500
C6A—C7A	1.401 (5)	C6B—C7B	1.415 (5)
C6A—H6A	0.9500	C6B—H6B	0.9500
C7A—C8A	1.373 (5)	C7B—C8B	1.378 (5)
C7A—H7A	0.9500	C7B—H7B	0.9500
C8A—N11A	1.396 (4)	C8B—N11B	1.410 (4)
C8A—C9A	1.423 (5)	C8B—C9B	1.431 (5)
C9A—C10A	1.422 (5)	C9B—C10B	1.408 (5)
N11A—C12A	1.360 (4)	N11B—C12B	1.364 (4)
N11A—H11A	0.74 (4)	N11B—H11B	0.90 (4)
C12A—O13A	1.241 (4)	C12B—O13B	1.230 (4)
C12A—N14A	1.357 (4)	C12B—N14B	1.353 (4)
N14A—C15A	1.403 (4)	N14B—C15B	1.407 (4)
N14A—H14A	0.86 (4)	N14B—H14B	0.80 (3)
C15A—C16A	1.389 (4)	C15B—C20B	1.394 (5)
C15A—C20A	1.400 (4)	C15B—C16B	1.394 (5)
C16A—C17A	1.377 (5)	C16B—C17B	1.384 (5)
C16A—H16A	0.9500	C16B—H16B	0.9500
C17A—C18A	1.396 (5)	C17B—C18B	1.390 (5)
C17A—H17A	0.9500	C17B—H17B	0.9500
C18A—C19A	1.389 (5)	C18B—C19B	1.396 (5)
C18A—C21A	1.511 (5)	C18B—C21B	1.513 (5)
C19A—C20A	1.385 (5)	C19B—C20B	1.381 (5)
C19A—H19A	0.9500	C19B—H19B	0.9500
C20A—H20A	0.9500	C20B—H20B	0.9500
C21A—C22A	1.511 (4)	C21B—C22B	1.515 (5)
C21A—H21A	0.9900	C21B—H21C	0.9900
C21A—H21B	0.9900	C21B—H21D	0.9900
C22A—C23A	1.377 (5)	C22B—C27B	1.368 (5)
C22A—C27A	1.393 (5)	C22B—C23B	1.400 (5)
C23A—C24A	1.380 (5)	C23B—C24B	1.378 (5)
C23A—H23A	0.9500	C23B—H23B	0.9500
C24A—C25A	1.391 (5)	C24B—C25B	1.375 (5)
C24A—H24A	0.9500	C24B—H24B	0.9500
C25A—C26A	1.381 (5)	C25B—C26B	1.392 (5)
C25A—N28A	1.412 (4)	C25B—N28B	1.417 (4)
C26A—C27A	1.384 (5)	C26B—C27B	1.386 (5)
C26A—H26A	0.9500	C26B—H26B	0.9500
C27A—H27A	0.9500	C27B—H27B	0.9500
N28A—C29A	1.346 (4)	N28B—C29B	1.355 (5)
N28A—H28A	0.78 (4)	N28B—H28B	0.87 (4)
C29A—O30A	1.234 (4)	C29B—O30B	1.234 (4)
C29A—N31A	1.366 (4)	C29B—N31B	1.361 (4)
N31A—C32A	1.392 (4)	N31B—C32B	1.402 (4)
N31A—H31A	0.88 (4)	N31B—H31B	0.90 (4)
C32A—C33A	1.374 (5)	C32B—C33B	1.379 (5)
C32A—C40A	1.435 (5)	C32B—C40B	1.430 (5)
C33A—C34A	1.413 (5)	C33B—C34B	1.412 (5)
C33A—H33A	0.9500	C33B—H33B	0.9500
C34A—C35A	1.363 (5)	C34B—C35B	1.365 (5)
C34A—H34A	0.9500	C34B—H34B	0.9500
C35A—C41A	1.414 (5)	C35B—C41B	1.413 (5)
C35A—H35A	0.9500	C35B—H35B	0.9500
C36A—C37A	1.357 (5)	C36B—C37B	1.362 (6)
C36A—C41A	1.421 (5)	C36B—C41B	1.405 (5)
C36A—H36A	0.9500	C36B—H36B	0.9500
C37A—C38A	1.409 (5)	C37B—C38B	1.405 (5)
C37A—H37A	0.9500	C37B—H37B	0.9500
C38A—N39A	1.324 (4)	C38B—N39B	1.321 (4)
C38A—H38A	0.9500	C38B—H38B	0.9500
N39A—C40A	1.368 (4)	N39B—C40B	1.376 (4)
C40A—C41A	1.409 (5)	C40B—C41B	1.408 (5)
			
C2A—N1A—C9A	116.9 (3)	C2B—N1B—C9B	116.8 (3)
N1A—C2A—C3A	123.9 (4)	N1B—C2B—C3B	123.4 (3)
N1A—C2A—H2A	118.0	N1B—C2B—H2B	118.3
C3A—C2A—H2A	118.0	C3B—C2B—H2B	118.3
C4A—C3A—C2A	119.1 (3)	C4B—C3B—C2B	119.6 (3)
C4A—C3A—H3A	120.4	C4B—C3B—H3B	120.2
C2A—C3A—H3A	120.4	C2B—C3B—H3B	120.2
C3A—C4A—C10A	119.7 (3)	C3B—C4B—C10B	119.6 (3)
C3A—C4A—H4A	120.2	C3B—C4B—H4B	120.2
C10A—C4A—H4A	120.2	C10B—C4B—H4B	120.2
C6A—C5A—C10A	120.1 (3)	C6B—C5B—C10B	119.1 (3)
C6A—C5A—H5A	120.0	C6B—C5B—H5B	120.5
C10A—C5A—H5A	120.0	C10B—C5B—H5B	120.5
C5A—C6A—C7A	121.7 (4)	C5B—C6B—C7B	122.6 (3)
C5A—C6A—H6A	119.2	C5B—C6B—H6B	118.7
C7A—C6A—H6A	119.2	C7B—C6B—H6B	118.7
C8A—C7A—C6A	119.9 (3)	C8B—C7B—C6B	119.1 (3)
C8A—C7A—H7A	120.0	C8B—C7B—H7B	120.4
C6A—C7A—H7A	120.0	C6B—C7B—H7B	120.4
C7A—C8A—N11A	125.8 (3)	C7B—C8B—N11B	125.1 (3)
C7A—C8A—C9A	119.8 (3)	C7B—C8B—C9B	120.1 (3)
N11A—C8A—C9A	114.3 (3)	N11B—C8B—C9B	114.8 (3)
N1A—C9A—C10A	123.6 (3)	N1B—C9B—C10B	124.0 (3)
N1A—C9A—C8A	117.4 (3)	N1B—C9B—C8B	117.0 (3)
C10A—C9A—C8A	118.9 (3)	C10B—C9B—C8B	119.0 (3)
C5A—C10A—C4A	123.7 (3)	C9B—C10B—C5B	120.1 (3)
C5A—C10A—C9A	119.5 (3)	C9B—C10B—C4B	116.6 (3)
C4A—C10A—C9A	116.7 (3)	C5B—C10B—C4B	123.4 (3)
C12A—N11A—C8A	129.2 (3)	C12B—N11B—C8B	126.5 (3)
C12A—N11A—H11A	114 (3)	C12B—N11B—H11B	122 (2)
C8A—N11A—H11A	117 (3)	C8B—N11B—H11B	110 (2)
O13A—C12A—N14A	123.4 (3)	O13B—C12B—N14B	123.4 (3)
O13A—C12A—N11A	123.4 (3)	O13B—C12B—N11B	123.1 (3)
N14A—C12A—N11A	113.2 (3)	N14B—C12B—N11B	113.5 (3)
C12A—N14A—C15A	127.3 (3)	C12B—N14B—C15B	125.2 (3)
C12A—N14A—H14A	116 (2)	C12B—N14B—H14B	119 (2)
C15A—N14A—H14A	115 (2)	C15B—N14B—H14B	115 (2)
C16A—C15A—C20A	118.7 (3)	C20B—C15B—C16B	119.2 (3)
C16A—C15A—N14A	118.0 (3)	C20B—C15B—N14B	121.8 (3)
C20A—C15A—N14A	123.2 (3)	C16B—C15B—N14B	118.8 (3)
C17A—C16A—C15A	121.2 (3)	C17B—C16B—C15B	119.7 (3)
C17A—C16A—H16A	119.4	C17B—C16B—H16B	120.1
C15A—C16A—H16A	119.4	C15B—C16B—H16B	120.1
C16A—C17A—C18A	121.0 (3)	C16B—C17B—C18B	121.9 (3)
C16A—C17A—H17A	119.5	C16B—C17B—H17B	119.1
C18A—C17A—H17A	119.5	C18B—C17B—H17B	119.1
C19A—C18A—C17A	117.3 (3)	C17B—C18B—C19B	117.6 (3)
C19A—C18A—C21A	122.1 (3)	C17B—C18B—C21B	122.0 (3)
C17A—C18A—C21A	120.5 (3)	C19B—C18B—C21B	120.4 (3)
C20A—C19A—C18A	122.6 (3)	C20B—C19B—C18B	121.5 (3)
C20A—C19A—H19A	118.7	C20B—C19B—H19B	119.3
C18A—C19A—H19A	118.7	C18B—C19B—H19B	119.3
C19A—C20A—C15A	119.2 (3)	C19B—C20B—C15B	120.1 (3)
C19A—C20A—H20A	120.4	C19B—C20B—H20B	120.0
C15A—C20A—H20A	120.4	C15B—C20B—H20B	120.0
C18A—C21A—C22A	114.1 (3)	C18B—C21B—C22B	114.5 (3)
C18A—C21A—H21A	108.7	C18B—C21B—H21C	108.6
C22A—C21A—H21A	108.7	C22B—C21B—H21C	108.6
C18A—C21A—H21B	108.7	C18B—C21B—H21D	108.6
C22A—C21A—H21B	108.7	C22B—C21B—H21D	108.6
H21A—C21A—H21B	107.6	H21C—C21B—H21D	107.6
C23A—C22A—C27A	117.4 (3)	C27B—C22B—C23B	117.8 (3)
C23A—C22A—C21A	120.4 (3)	C27B—C22B—C21B	122.4 (3)
C27A—C22A—C21A	122.1 (3)	C23B—C22B—C21B	119.8 (3)
C22A—C23A—C24A	122.4 (3)	C24B—C23B—C22B	120.4 (3)
C22A—C23A—H23A	118.8	C24B—C23B—H23B	119.8
C24A—C23A—H23A	118.8	C22B—C23B—H23B	119.8
C23A—C24A—C25A	119.5 (3)	C25B—C24B—C23B	121.0 (3)
C23A—C24A—H24A	120.2	C25B—C24B—H24B	119.5
C25A—C24A—H24A	120.2	C23B—C24B—H24B	119.5
C26A—C25A—C24A	119.1 (3)	C24B—C25B—C26B	119.3 (3)
C26A—C25A—N28A	118.3 (3)	C24B—C25B—N28B	118.2 (3)
C24A—C25A—N28A	122.6 (3)	C26B—C25B—N28B	122.5 (3)
C25A—C26A—C27A	120.5 (3)	C27B—C26B—C25B	118.8 (3)
C25A—C26A—H26A	119.8	C27B—C26B—H26B	120.6
C27A—C26A—H26A	119.8	C25B—C26B—H26B	120.6
C26A—C27A—C22A	121.1 (3)	C22B—C27B—C26B	122.6 (3)
C26A—C27A—H27A	119.5	C22B—C27B—H27B	118.7
C22A—C27A—H27A	119.5	C26B—C27B—H27B	118.7
C29A—N28A—C25A	126.0 (3)	C29B—N28B—C25B	126.0 (3)
C29A—N28A—H28A	120 (3)	C29B—N28B—H28B	118 (3)
C25A—N28A—H28A	114 (3)	C25B—N28B—H28B	113 (3)
O30A—C29A—N28A	123.9 (3)	O30B—C29B—N28B	123.5 (3)
O30A—C29A—N31A	122.5 (3)	O30B—C29B—N31B	123.6 (3)
N28A—C29A—N31A	113.6 (3)	N28B—C29B—N31B	112.9 (3)
C29A—N31A—C32A	128.2 (3)	C29B—N31B—C32B	128.4 (3)
C29A—N31A—H31A	120 (2)	C29B—N31B—H31B	121 (2)
C32A—N31A—H31A	112 (2)	C32B—N31B—H31B	110 (2)
C33A—C32A—N31A	126.0 (3)	C33B—C32B—N31B	125.5 (3)
C33A—C32A—C40A	119.3 (3)	C33B—C32B—C40B	119.2 (3)
N31A—C32A—C40A	114.7 (3)	N31B—C32B—C40B	115.3 (3)
C32A—C33A—C34A	120.4 (3)	C32B—C33B—C34B	120.0 (3)
C32A—C33A—H33A	119.8	C32B—C33B—H33B	120.0
C34A—C33A—H33A	119.8	C34B—C33B—H33B	120.0
C35A—C34A—C33A	121.1 (4)	C35B—C34B—C33B	121.6 (3)
C35A—C34A—H34A	119.4	C35B—C34B—H34B	119.2
C33A—C34A—H34A	119.4	C33B—C34B—H34B	119.2
C34A—C35A—C41A	120.2 (3)	C34B—C35B—C41B	119.9 (3)
C34A—C35A—H35A	119.9	C34B—C35B—H35B	120.0
C41A—C35A—H35A	119.9	C41B—C35B—H35B	120.0
C37A—C36A—C41A	119.1 (4)	C37B—C36B—C41B	119.5 (3)
C37A—C36A—H36A	120.4	C37B—C36B—H36B	120.3
C41A—C36A—H36A	120.5	C41B—C36B—H36B	120.3
C36A—C37A—C38A	119.6 (3)	C36B—C37B—C38B	119.6 (4)
C36A—C37A—H37A	120.2	C36B—C37B—H37B	120.2
C38A—C37A—H37A	120.2	C38B—C37B—H37B	120.2
N39A—C38A—C37A	123.8 (4)	N39B—C38B—C37B	123.4 (4)
N39A—C38A—H38A	118.1	N39B—C38B—H38B	118.3
C37A—C38A—H38A	118.1	C37B—C38B—H38B	118.3
C38A—N39A—C40A	116.8 (3)	C38B—N39B—C40B	117.0 (3)
N39A—C40A—C41A	123.5 (3)	N39B—C40B—C41B	123.2 (3)
N39A—C40A—C32A	116.8 (3)	N39B—C40B—C32B	116.6 (3)
C41A—C40A—C32A	119.6 (3)	C41B—C40B—C32B	120.2 (3)
C40A—C41A—C35A	119.4 (3)	C36B—C41B—C40B	117.2 (3)
C40A—C41A—C36A	117.1 (3)	C36B—C41B—C35B	123.6 (3)
C35A—C41A—C36A	123.5 (3)	C40B—C41B—C35B	119.2 (3)
			
C9A—N1A—C2A—C3A	0.9 (5)	C9B—N1B—C2B—C3B	−1.2 (5)
N1A—C2A—C3A—C4A	0.5 (6)	N1B—C2B—C3B—C4B	−0.2 (5)
C2A—C3A—C4A—C10A	−1.1 (6)	C2B—C3B—C4B—C10B	0.3 (5)
C10A—C5A—C6A—C7A	0.9 (6)	C10B—C5B—C6B—C7B	−0.4 (5)
C5A—C6A—C7A—C8A	−0.3 (6)	C5B—C6B—C7B—C8B	0.5 (5)
C6A—C7A—C8A—N11A	179.1 (3)	C6B—C7B—C8B—N11B	179.7 (3)
C6A—C7A—C8A—C9A	−0.1 (5)	C6B—C7B—C8B—C9B	−0.7 (5)
C2A—N1A—C9A—C10A	−1.9 (5)	C2B—N1B—C9B—C10B	2.5 (5)
C2A—N1A—C9A—C8A	176.4 (3)	C2B—N1B—C9B—C8B	−178.7 (3)
C7A—C8A—C9A—N1A	−178.4 (3)	C7B—C8B—C9B—N1B	−178.1 (3)
N11A—C8A—C9A—N1A	2.3 (4)	N11B—C8B—C9B—N1B	1.5 (4)
C7A—C8A—C9A—C10A	0.0 (5)	C7B—C8B—C9B—C10B	0.8 (5)
N11A—C8A—C9A—C10A	−179.4 (3)	N11B—C8B—C9B—C10B	−179.6 (3)
C6A—C5A—C10A—C4A	176.4 (4)	N1B—C9B—C10B—C5B	178.2 (3)
C6A—C5A—C10A—C9A	−1.0 (6)	C8B—C9B—C10B—C5B	−0.6 (5)
C3A—C4A—C10A—C5A	−177.2 (4)	N1B—C9B—C10B—C4B	−2.3 (5)
C3A—C4A—C10A—C9A	0.3 (5)	C8B—C9B—C10B—C4B	178.9 (3)
N1A—C9A—C10A—C5A	178.8 (3)	C6B—C5B—C10B—C9B	0.5 (5)
C8A—C9A—C10A—C5A	0.6 (5)	C6B—C5B—C10B—C4B	−179.0 (3)
N1A—C9A—C10A—C4A	1.3 (5)	C3B—C4B—C10B—C9B	0.8 (5)
C8A—C9A—C10A—C4A	−177.0 (3)	C3B—C4B—C10B—C5B	−179.6 (3)
C7A—C8A—N11A—C12A	6.3 (6)	C7B—C8B—N11B—C12B	−18.5 (5)
C9A—C8A—N11A—C12A	−174.5 (3)	C9B—C8B—N11B—C12B	161.9 (3)
C8A—N11A—C12A—O13A	2.6 (6)	C8B—N11B—C12B—O13B	−0.4 (5)
C8A—N11A—C12A—N14A	−178.5 (3)	C8B—N11B—C12B—N14B	−179.2 (3)
O13A—C12A—N14A—C15A	−5.6 (5)	O13B—C12B—N14B—C15B	−9.0 (5)
N11A—C12A—N14A—C15A	175.5 (3)	N11B—C12B—N14B—C15B	169.8 (3)
C12A—N14A—C15A—C16A	−150.1 (3)	C12B—N14B—C15B—C20B	−33.3 (5)
C12A—N14A—C15A—C20A	33.2 (5)	C12B—N14B—C15B—C16B	152.2 (3)
C20A—C15A—C16A—C17A	−3.7 (5)	C20B—C15B—C16B—C17B	3.0 (5)
N14A—C15A—C16A—C17A	179.5 (3)	N14B—C15B—C16B—C17B	177.6 (3)
C15A—C16A—C17A—C18A	1.2 (5)	C15B—C16B—C17B—C18B	−0.3 (5)
C16A—C17A—C18A—C19A	1.5 (5)	C16B—C17B—C18B—C19B	−1.8 (5)
C16A—C17A—C18A—C21A	−176.0 (3)	C16B—C17B—C18B—C21B	176.5 (3)
C17A—C18A—C19A—C20A	−1.7 (5)	C17B—C18B—C19B—C20B	1.2 (5)
C21A—C18A—C19A—C20A	175.8 (3)	C21B—C18B—C19B—C20B	−177.1 (3)
C18A—C19A—C20A—C15A	−0.8 (5)	C18B—C19B—C20B—C15B	1.4 (5)
C16A—C15A—C20A—C19A	3.5 (5)	C16B—C15B—C20B—C19B	−3.5 (5)
N14A—C15A—C20A—C19A	−179.9 (3)	N14B—C15B—C20B—C19B	−177.9 (3)
C19A—C18A—C21A—C22A	118.7 (4)	C17B—C18B—C21B—C22B	37.5 (5)
C17A—C18A—C21A—C22A	−63.9 (4)	C19B—C18B—C21B—C22B	−144.3 (3)
C18A—C21A—C22A—C23A	152.7 (3)	C18B—C21B—C22B—C27B	−116.6 (4)
C18A—C21A—C22A—C27A	−29.9 (5)	C18B—C21B—C22B—C23B	64.9 (4)
C27A—C22A—C23A—C24A	−1.0 (5)	C27B—C22B—C23B—C24B	−2.7 (5)
C21A—C22A—C23A—C24A	176.5 (3)	C21B—C22B—C23B—C24B	175.8 (3)
C22A—C23A—C24A—C25A	−1.3 (5)	C22B—C23B—C24B—C25B	−0.4 (6)
C23A—C24A—C25A—C26A	2.5 (5)	C23B—C24B—C25B—C26B	3.1 (6)
C23A—C24A—C25A—N28A	178.8 (3)	C23B—C24B—C25B—N28B	−178.5 (3)
C24A—C25A—C26A—C27A	−1.4 (5)	C24B—C25B—C26B—C27B	−2.7 (5)
N28A—C25A—C26A—C27A	−177.8 (3)	N28B—C25B—C26B—C27B	179.0 (3)
C25A—C26A—C27A—C22A	−0.9 (6)	C23B—C22B—C27B—C26B	3.1 (5)
C23A—C22A—C27A—C26A	2.1 (5)	C21B—C22B—C27B—C26B	−175.4 (3)
C21A—C22A—C27A—C26A	−175.4 (3)	C25B—C26B—C27B—C22B	−0.5 (5)
C26A—C25A—N28A—C29A	−154.3 (4)	C24B—C25B—N28B—C29B	144.2 (4)
C24A—C25A—N28A—C29A	29.5 (6)	C26B—C25B—N28B—C29B	−37.5 (6)
C25A—N28A—C29A—O30A	6.7 (6)	C25B—N28B—C29B—O30B	8.3 (6)
C25A—N28A—C29A—N31A	−172.7 (3)	C25B—N28B—C29B—N31B	−172.3 (3)
O30A—C29A—N31A—C32A	−6.0 (6)	O30B—C29B—N31B—C32B	2.1 (6)
N28A—C29A—N31A—C32A	173.4 (3)	N28B—C29B—N31B—C32B	−177.2 (3)
C29A—N31A—C32A—C33A	6.9 (6)	C29B—N31B—C32B—C33B	−2.3 (6)
C29A—N31A—C32A—C40A	−171.6 (3)	C29B—N31B—C32B—C40B	176.9 (3)
N31A—C32A—C33A—C34A	−177.9 (3)	N31B—C32B—C33B—C34B	178.7 (3)
C40A—C32A—C33A—C34A	0.5 (5)	C40B—C32B—C33B—C34B	−0.4 (5)
C32A—C33A—C34A—C35A	0.0 (5)	C32B—C33B—C34B—C35B	−0.3 (5)
C33A—C34A—C35A—C41A	0.0 (6)	C33B—C34B—C35B—C41B	0.5 (5)
C41A—C36A—C37A—C38A	−0.1 (6)	C41B—C36B—C37B—C38B	1.3 (6)
C36A—C37A—C38A—N39A	0.3 (6)	C36B—C37B—C38B—N39B	−1.6 (6)
C37A—C38A—N39A—C40A	0.8 (5)	C37B—C38B—N39B—C40B	0.4 (5)
C38A—N39A—C40A—C41A	−2.1 (5)	C38B—N39B—C40B—C41B	1.0 (5)
C38A—N39A—C40A—C32A	177.6 (3)	C38B—N39B—C40B—C32B	−179.0 (3)
C33A—C32A—C40A—N39A	179.3 (3)	C33B—C32B—C40B—N39B	−179.0 (3)
N31A—C32A—C40A—N39A	−2.1 (4)	N31B—C32B—C40B—N39B	1.8 (4)
C33A—C32A—C40A—C41A	−1.0 (5)	C33B—C32B—C40B—C41B	1.0 (5)
N31A—C32A—C40A—C41A	177.7 (3)	N31B—C32B—C40B—C41B	−178.2 (3)
N39A—C40A—C41A—C35A	−179.4 (3)	C37B—C36B—C41B—C40B	0.0 (5)
C32A—C40A—C41A—C35A	0.9 (5)	C37B—C36B—C41B—C35B	179.5 (4)
N39A—C40A—C41A—C36A	2.2 (5)	N39B—C40B—C41B—C36B	−1.2 (5)
C32A—C40A—C41A—C36A	−177.5 (3)	C32B—C40B—C41B—C36B	178.8 (3)
C34A—C35A—C41A—C40A	−0.4 (5)	N39B—C40B—C41B—C35B	179.3 (3)
C34A—C35A—C41A—C36A	177.8 (4)	C32B—C40B—C41B—C35B	−0.7 (5)
C37A—C36A—C41A—C40A	−1.0 (5)	C34B—C35B—C41B—C36B	−179.5 (3)
C37A—C36A—C41A—C35A	−179.4 (4)	C34B—C35B—C41B—C40B	0.0 (5)

### Hydrogen-bond geometry (Å, °)

**Table:**

*D*—H···*A*	*D*—H	H···*A*	*D*···*A*	*D*—H···*A*
N11A—H11A···N1A	0.74 (4)	2.27 (4)	2.625 (4)	110 (3)
N11A—H11A···O13A^i^	0.74 (4)	2.41 (4)	3.101 (3)	155 (4)
N14A—H14A···O13A^i^	0.86 (4)	1.97 (4)	2.810 (4)	166 (3)
N28A—H28A···O30A^ii^	0.78 (4)	2.05 (4)	2.827 (4)	170 (4)
N31A—H31A···O30A^ii^	0.88 (4)	2.56 (4)	3.293 (4)	141 (3)
N31A—H31A···N39A	0.88 (4)	2.14 (4)	2.635 (4)	114 (3)
N11B—H11B···N1B	0.90 (4)	2.13 (4)	2.645 (4)	116 (3)
N11B—H11B···O30B^iii^	0.90 (4)	2.46 (4)	3.172 (4)	136 (3)
N14B—H14B···O30B^iii^	0.80 (3)	1.98 (4)	2.772 (4)	167 (3)
N28B—H28B···O13B^iv^	0.87 (4)	1.94 (4)	2.786 (4)	161 (4)
N31B—H31B···O13B^iv^	0.90 (4)	2.36 (3)	3.115 (4)	141 (3)
N31B—H31B···N39B	0.90 (4)	2.14 (3)	2.647 (4)	115 (3)

Symmetry codes: (i) *y*, −*x*+1, *z*+1/4; (ii) *y*+1, −*x*+1, *z*+1/4; (iii) −*y*+1, *x*−1, *z*−1/4; (iv) −*y*+1, *x*, *z*−1/4.
